# Confirming putative variants at ≤ 5% allele frequency using allele enrichment and Sanger sequencing

**DOI:** 10.1038/s41598-021-91142-1

**Published:** 2021-06-02

**Authors:** Yan Helen Yan, Sherry X. Chen, Lauren Y. Cheng, Alyssa Y. Rodriguez, Rui Tang, Karina Cabrera, David Yu Zhang

**Affiliations:** 1 NuProbe USA, Inc., Houston, TX USA; 2 Department of Bioengineering, Rice University, 6500 Main St, Houston, TX 77030 USA; 3 Systems, Synthetic, and Physical Biology, Rice University, 6500 Main St, Houston, TX 77030 USA

**Keywords:** Next-generation sequencing, Assay systems, DNA sequencing, Thermodynamics, DNA probes, Oligonucleotide probes, PCR-based techniques

## Abstract

Whole exome sequencing (WES) is used to identify mutations in a patient’s tumor DNA that are predictive of tumor behavior, including the likelihood of response or resistance to cancer therapy. WES has a mutation limit of detection (LoD) at variant allele frequencies (VAF) of 5%. Putative mutations called at ≤ 5% VAF are frequently due to sequencing errors, therefore reporting these subclonal mutations incurs risk of significant false positives. Here we performed ~ 1000 × WES on fresh-frozen and formalin-fixed paraffin-embedded (FFPE) tissue biopsy samples from a non-small cell lung cancer patient, and identified 226 putative mutations at between 0.5 and 5% VAF. Each variant was then tested using NuProbe NGSure, to confirm the original WES calls. NGSure utilizes Blocker Displacement Amplification to first enrich the allelic fraction of the mutation and then uses Sanger sequencing to determine mutation identity. Results showed that 52% of the 226 (117) putative variants were disconfirmed, among which 2% (5) putative variants were found to be misidentified in WES. In the 66 cancer-related variants, the disconfirmed rate was 82% (54/66). This data demonstrates Blocker Displacement Amplification allelic enrichment coupled with Sanger sequencing can be used to confirm putative mutations ≤ 5% VAF. By implementing this method, next-generation sequencing can reliably report low-level variants at a high sensitivity, without the cost of high sequencing depth.

## Introduction

Next-generation sequencing (NGS) quickly emerges as a primary clinical diagnostic platform. One use of NGS is whole exome sequencing (WES), in which the entire exome is enriched and sequenced to detect disease-causing variants^1^. The exon region is roughly 1% of the human genome and responsible for tumor-causing mutations that lead to altered protein functions^2^. WES has also been used to characterize mutational signatures across tumor types^3^.

**Figure 1:**
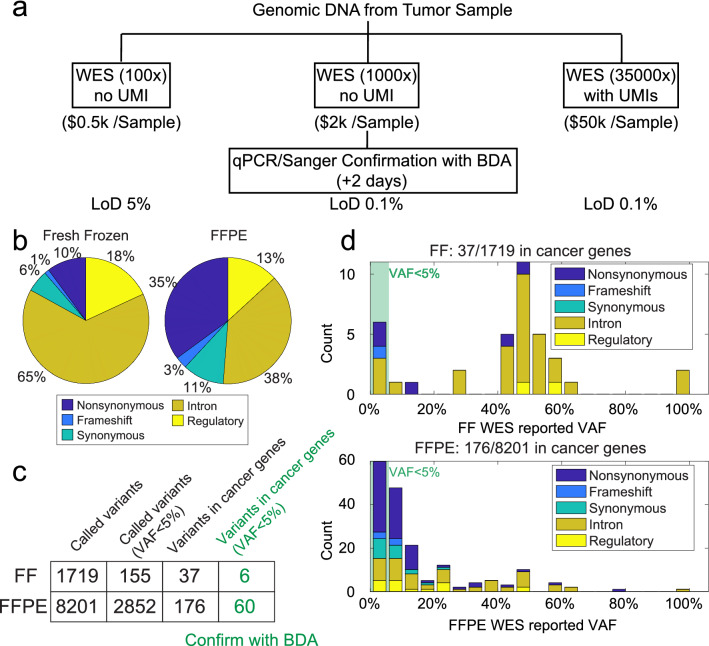
Somatic variant detection by whole exome sequencing of non-small cell lung cancer tumor samples. (**a**) Different depths of WES have different costs and LoDs. By incorporating BDA methodology, WES can achieve 0.1% VAF LoD at lower cost. (**b**) Categorical breakdown of WES detected somatic variants in the fresh-frozen (FF) sample (n = 1719) and the FFPE sample (n = 8201). (**c**) Number of somatic variants called by WES. All 66 variants with < 5% VAF and in cancer-related genes underwent confirmatory analysis by BDA. Another 160 variants with < 5% VAF but not in cancer-related genes also underwent confirmatory analysis by BDA. (**d**) VAF histogram and categorical breakdown of somatic variants detected in cancer-related genes.

For NGS panels like WES, intrinsic sequencing error rate and sequencing depth are bottlenecks for detection of low-frequency variants. In most laboratories, WES is performed at 100 × for somatic mutation discovery, which costs ~ $500 with an LoD of 5–10% VAF (Fig. 1a)^4,5^. For many tumor sequencing applications, 5% VAF mutation sensitivity is sufficient. However, higher mutation sensitivity is required to identify subclonal drug resistance mutations from tumor tissue or perform non-invasive tumor profiling with cell-free DNA from peripheral blood samples (“liquid biopsy”)^6,7^. For these applications, high clinical sensitivity can only be achieved when the NGS panel's mutation sensitivity reaches 0.1% to 0.5% VAF. To reliably make mutation calls at 0.1% VAF, WES at 35,000 × utilizing Unique Molecular Identifiers is needed (UMIs; e.g., 70-gene Guardant 360 panel^8,9^ and 500-gene Illumina TruSight panel^10^). But at a price of $50 k per sample^7,11,12^, it is impractical for many laboratories to use ultra-deep NGS assays.

Additionally, WES is susceptible to high false-positive rates caused by variant calling errors. This is due to limitation of sequencing depth, NGS intrinsic error, gene coverage nonuniformity, and errors from variant calling software^4,5^. Belkadi et al. reported that the proportion of false-positive variants was as high as 78% for single-nucleotide variants (SNVs) and 44% for indels in WES assessed by Sanger sequencing^13^. Therefore, there is a strong need for orthogonal variant confirmation.

Sanger sequencing, also known as the “chain termination method”, is a fast, cost-effective sequencing method that has been used in this field for more than 40 years^14^. Sanger sequencing is considered the gold standard for pathology laboratories due to its robustness and well-established nature^15,16^. Mu et al. reported an extensive Sanger confirmation of 7845 variants called in a 14-gene NGS panel; nearly all disconfirmed variants were concentrated at VAF < 20%^15^. However, because Sanger sequencing has an LoD of 5–20% VAF, it cannot be used directly to confirm NGS variant findings with VAF < 5%.

Another method used for low-level variant detection is Blocker Displacement Amplification technology (BDA). BDA technology is a novel PCR-based enrichment method for preferential amplification of low-level variants over the wildtype sequence^17^. BDA was validated for use in WES confirmation of rare variants, providing an LoD of 0.1% VAF to determine if mutations found in child proband by WES were de novo or parental somatic mosaicism mutations^18^. The results were compared with droplet digital PCR (ddPCR), and the obtained VAFs were similar. Sanger sequencing coupled with BDA technology was also used to detect low-level PIK3CA mutations in melanoma tumor sections^19^, to estimate the VAF of low-level mosaic mutations in families with alveolar capillary dysplasia^20^, and to detect BRAF mutations down to 0.2% VAF in FFPE lymph node tissue samples from metastatic melanoma patients^21^. In addition, BDA was used in multiplex PCR and coupled with amplicon sequencing for the detection of low-level variants in NGS as well^22^.

Here we present an orthogonal method of confirming low VAF mutations called in WES utilizing Sanger sequencing incorporating BDA technology. As an auxiliary step following WES, BDA enriches the low-level variant to dominant level on Sanger trace for confirmation, which dramatically increases the specificity of WES variant detection without the need for extreme depth. We performed a 1000 × WES without UMIs on a mirrored pair of fresh-frozen and formalin-fixed paraffin-embedded (FFPE) tumor samples from a patient with Stage IIA non-small cell lung cancer (NSCLC) at $2 k/sample. Of the called variants below 5% VAF, we selected 226 and performed BDA qPCR/Sanger confirmation analysis.

## Materials and methods

### Samples and study materials

A mirrored pair of fresh-frozen and FFPE tissue blocks from a single stage IIA lung adenocarcinoma (AJCC Stage T2aN1M0) patient was purchased from BioIVT. The samples were collected by BioIVT with written informed patient consent. IRB approval was not required as these are Exemption 4 (commercial, de-identified samples). Based on histological studies, tumor occupies 70% of both tissue blocks. Serial sections were cut at 10 μm from the blocks and collected in separate tubes. For the following experiments, 12 fresh-frozen sections and 12 FFPE sections were used to extract the DNA.

### DNA extraction from fresh-frozen and FFPE samples

The QIAamp DNA mini kit (Qiagen, 51304) was used on fresh-frozen sample to perform DNA extraction, while GeneRead DNA FFPE Kit (Qiagen, 180134) was used on FFPE sample. The NEBNext FFPE DNA Repair Mix (New England Biolabs, M6630S) was used to repair extracted FFPE DNA. NanoDrop spectrophotometer and Qubit Fluorometer were used to measure yield of DNA. Quality control was done via Bioanalyzer. DNA materials were stored at − 20 °C until ready for analysis. Whole exome sequencing, BDA qPCR and Sanger analysis all used samples from the same DNA extract.

### Whole exome sequencing

Fresh-frozen and FFPE genomic DNA (1 μg each) were sent to GENEWIZ for deep whole exome sequencing using Agilent SureSelect Human All Exon V6 capture panel. Each sample was sequenced using one Illumina HiSeq lane. The mean bait depth for fresh-frozen sample and FFPE sample were 1289 × and 1067 × .

### Whole exome sequencing variant analysis

Somatic variant calling was performed by GENEWIZ bioinformatics solutions. SNVs and small indels were called using the Dragen somatic pipeline. A “panel of normal” made from > 50 unrelated normal samples was used to remove recurrent technical artifacts. The VCF output generated by the pipeline was then normalized using BCFtools. Variants marked as common in dbSNP build 151 were filtered out. The filtered VCF output was then annotated with Ensembl VEP v95. Overall, 1719 somatic variants from fresh-frozen sample and 8201 from FFPE sample were called.

### Candidate variants selection

We selected 94 putative variants from fresh-frozen sample and 132 from FFPE sample with < 5% WES VAF for BDA confirmation analysis. All < 5% VAF variants that were in the FoundationOne CDx gene list (Foundation Medicine) were selected, which include 6 variants in fresh-frozen sample and 60 in FFPE sample. Another 160 low-level variants were selected randomly.

### Custom BDA assay development

We developed a custom BDA assay for each selected variant. Algorithmic design of BDA oligos (primers and blockers) was performed by the NGSure software platform (NuProbe). This platform designs primer and blocker sequences based on BDA principles described in detail by Wu et al.^17^. The designed sequences were ordered from Integrated DNA Technologies as standard oligos.

Analytical validation of BDA assays was performed by testing each assay on a negative control and a positive control. The negative control was wildtype genomic DNA (Coriell, NA18537). The positive control was synthetic gBlocks containing the respective variant ordered from IDT. Synthetic gBlocks were diluted to approximately 3000 molecules/μl, and concentrations were estimated by qPCR. The Cq values of the synthetic gBlocks were compared to Cq values of 10 ng per well genomic DNA assayed with the same primers. The concentrations of the synthetic templates were then adjusted to ensure that positive and negative controls had same amplifiable copy number. 10 ng gDNA (0% VAF negative control) and equivalent synthetic gBlock (100% VAF positive control) were then tested on each BDA assay. Each assay was validated to have greater than 10 Cq difference between negative and positive controls before proceeding to real sample testing.

### qPCR/Sanger confirmation of putative variants

We performed 94 confirmation assays for fresh-frozen and 132 for FFPE following the NGSure Custom qPCR/Sanger Variant Assay (NuProbe) user manual. For each variant, the sample was tested with blocker (i.e., standard BDA to enrich for variant of interest) and without blocker (i.e., forward and reverse primers only, for input quantification and normalization). PowerUp SYBR Green Master Mix (Thermo Fisher Scientific) was used with 400 nM primer, 4 µM blocker, and 10 ng of DNA per well for qPCR assays with a final volume of 10 µL. CFX96 Touch Real-Time PCR Detection System (Bio-Rad) with incubation at 95 °C for 180 s followed by 45 cycles of 95 °C for 10 s and 60 °C for 30 s was used to conduct all reactions. Each reaction was duplicated or triplicated.

BDA qPCR products were sent to GENEWIZ for Sanger Sequencing. Ab1 files were read by A Plasmic Editor (ApE) software, and variant status was visually inspected by comparing Sanger traces with reference sequences at loci of interest. If loci of interest matched neither wildtype nor putative variants, the reactions were repeated at least twice to eliminate any random effect.

∆*Cq* values were calculated for each variant using *Cq* values obtained in each of the two experiments:

1 $$\Delta Cq \,samp\mathrm{le}=\left(with\_blocker Cq\right)-(no\_blocker Cq)$$

A smaller *∆Cq sample* indicates a higher probability of the sample containing a variant.

If the presence of a variant allele was confirmed by Sanger sequencing, the qPCR ∆*Cq* values were used to estimate the theoretical VAF. Theoretical VAF was estimated as follows:

2 $$VAF= \frac{100\%}{{2}^{\Delta Cq \,sample} -{ 2}^{\Delta Cq \,gBlock}}$$

where *∆ Cq sample* is the ∆Cq of fresh-frozen or FFPE sample, and *∆ Cq gBlock* is the ∆Cq of the corresponding gBlock positive control for the respective variant.

We calculated the VAF based on gBlock 100% and assumed PCR amplification efficiency for mutant was 2 per cycle in reactions containing blocker, so that the Cq difference between sample and gBlock could be used to infer VAF in the sample. The quantitative accuracy of this method is within 1.5-fold difference compared to ddPCR^18^.

### Amplicon sequencing

There are three possible results for a qPCR/Sanger confirmation: variant confirmed, variant absent, and variant misidentified. For all variant misidentified cases, we constructed a targeted NGS library with primers covering the putative variant regions to further verify the base identity detected by BDA plus Sanger sequencing. We sequenced the library on MiniSeq (Illumina Inc.) with a median of 5000 reads for each amplicon. Integrative Genomics Viewer (IGV v2.8.9) software^23^ was used to analyze the data, as well as in-house developed scripts.

## Results

### Whole exome sequencing of non-small cell lung cancer tumor samples

We performed WES on fresh-frozen sample at an average of 1289 × and on FFPE sample at 1067 × average. Illumina DRAGEN somatic variant caller was used to identify small variants and indels^24^. WES identified 1719 somatic variants in the fresh-frozen sample and 8201 somatic variants in the FFPE sample. Between the two samples, 1111 variants were concordant. The distribution of variants depended on sample type (fresh-frozen vs. FFPE). Nonsynonymous or frameshift variants that lead to altered amino acid sequence and protein function made up 11% of putative variants in fresh-frozen sample and 38% in the FFPE sample (Fig. 1b). There were 155 variants in the fresh-frozen sample and 2852 in the FFPE sample that were low-level variants with less than 5% VAF (Fig. 1c).

Cancer exome sequencing usually reports somatic sequence variants in genes with diagnostic, prognostic, or predictive clinical evidence or in genes from cancer pathways, gene families, or functional groups that are therapeutic agents’ targets. The FoundationOne CDx panel provides a comprehensive list of 324 such genes^25^. In this manuscript, we define cancer-related genes to be those included in the FoundationOne CDx panel. Among all WES-detected variants, 37 from fresh-frozen sample and 176 from FFPE sample were found in cancer-related genes; of those, 6 in fresh-frozen sample and 60 in FFPE sample were low-level variants having VAF < 5% (Fig. 1c). In low-level variants from cancer-related genes, there were 2 nonsynonymous and 1 frameshift variant in fresh-frozen sample and 33 nonsynonymous and 3 frameshift variants in FFPE sample (Fig. 1d). The reason that FFPE sample showed more low-level variants than fresh-frozen is perhaps due to DNA damage occurring during fixation and storage, such as cytosine deamination and oxidation^26–28^. Deamination of nucleotides causes C:G > T:A changes while oxidative DNA damage causes C:G > A:T changes. Although DNA extracted from FFPE sample was enzymatically repaired before experiments, unrepaired DNA damages may still present at a low-level and be detected as base changes. For those 66 low-level cancer-related variants in fresh-frozen or FFPE samples, we further examined their presence with BDA qPCR/Sanger analysis, along with another 160 randomly selected low-level variants.

### BDA qPCR/Sanger confirmation of WES-called putative variants with VAF < 5%

Among the < 5% VAF variants, we selected 94 from fresh-frozen sample and 132 from FFPE sample for confirmatory BDA qPCR/Sanger analysis, including all WES-detected low-level variants (6 in fresh-frozen, 60 in FFPE sample) of cancer-related genes from the FoundationOne CDx list. The lowest VAF among all WES-called variants was 0.5%; thus, because BDA assays have an analytical LoD of 0.1%, putative variants were examined at 0.5–5% VAF.

**Figure 2:**
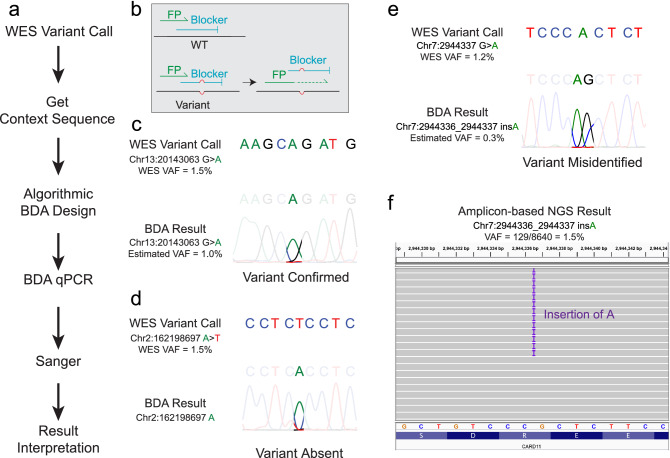
WES variant confirmation using Blocker Displacement Amplification (BDA). (**a**) Workflow for BDA confirmation of WES variants. (**b**) Mechanism for BDA variant enrichment^16^. The blocker preferentially binds to wildtype (WT) DNA sequences and suppresses their PCR amplification, resulting in selective amplification of variants. FP, forward primer. (**c**) Example 1: WES-called variant confirmed by BDA. (**d**) Example 2: Locus of interest was identified as wildtype by BDA, indicating that the variant called by WES was absent in the sample. (**e**) Example 3: Locus of interest showing a peak with an insertion rather than the WES-called substitution, indicating that the variant was misidentified by WES. (**f**) The amplicon-based NGS result (Integrative Genomics Viewer) for variants in (**e**) matches the BDA result.

NGSure BDA designer takes the input of the chromosome position and considers bioinformatic features such as amplicon GC content, the presence of pseudogenes and/or common germline SNPs near the priming/blocking regions (Fig. 2a). A BDA design contains a forward primer, a reverse primer and a blocker. The blocker is designed to be complementary to the known template sequence and contains an overlap region with the forward primer, forcing competition for binding. In templates containing variant, there is a resulting mismatch bubble when the blocker binds to the variant region. This weak binding allows the primer to displace the blocker and thereby promotes selective amplification of the variant sequence. This results in the differential amplification of the variant, yielding over 1000-fold enrichment when compounded over multiple cycles^17^ (Fig. 2b).

Figure 2c–e shows representative Sanger traces for 3 putative variants where customized BDA assays were applied. If the sequence of the locus of interest matched the WES call, the variant was confirmed (Fig. 2c). In this case, qPCR Cq values were used to estimate the VAF of the variant. If the sequence at the locus of interest matched the wildtype, the variant was disconfirmed (variant absent in the sample). If the sequence matched neither the wildtype nor the WES-called variant, the experiment was repeated at least twice to ensure the result accuracy. Figure 2e shows a case where the WES-called variant was Chr7:2944337 G > A, while Sanger sequencing of BDA amplicon revealed an *insertion* of base A instead of *substitution* of base A, indicating the variant was misidentified by WES. Of the 226 variants undergoing BDA analysis, 5 were classified as variants misidentified by WES. We examined these 5 variants using amplicon-based NGS with a median 5000 × depth per amplicon and all 5 variants were validated to be concordant with BDA analysis. Figure 2f shows the IGV results validating the Chr7:2944336_2944337 insA variant detected by BDA Sanger sequencing (Fig. 2e). For all WES-misidentified variants, Sanger traces from triplicate BDA reactions and IGV pileups of amplicon-based NGS reads are presented in Supplementary Fig. S3. Note that NGSure custom assays do not need further verification by amplicon-based sequencing; BDA qPCR/Sanger is sufficiently accurate. Amplicon-based sequencing experiments in this study were used only to validate our method.

**Figure 3:**
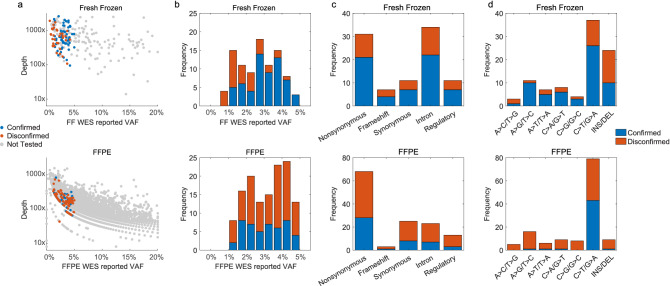
Characterization of WES variant confirmation. (**a**) The confirmation profile was determined by plotting the WES depths against the VAFs. We tested 94 variants called from the fresh-frozen sample and 132 variants called from the FFPE sample by BDA qPCR/Sanger analysis. Variants were either confirmed (blue) or disconfirmed (red). Variants called in WES but not tested by BDA are shown in grey. (**b**) BDA confirmation results by VAF distribution. (**c**) BDA confirmation results by variant functional categories. (**d**) BDA confirmation results by alteration types.

For loci where somatic variants are detected, the mean depth was 527 × in fresh-frozen sample and 131 × in FFPE sample (Fig. 3a). Of the 155 somatic variants in fresh-frozen sample and 2852 somatic variants in FFPE sample with < 5% VAF in WES, the mean depth was 824 × for fresh-frozen sample (ranging from 95 × to 2585 ×) and 243 × for FFPE sample (1 × to 1570 ×). Although both samples were sequenced for more than 1000 × , the actual sequencing depth of FFPE-detected variants is significantly less. This may be caused by the uneven coverage of FFPE-derived DNA, primarily attributed to short fragment length. For fresh-frozen sample, 842 M raw reads were obtained, where 535 M (63.7%) were unique reads and target bases above 20 × were 97.3%. For FFPE sample, 857 M raw reads were obtained, where 181 M (21.1%) were unique reads and target bases above 20 × were 71.9%.

Among the 226 WES-detected low-level variants undergoing confirmatory BDA qPCR/Sanger analysis, the overall disconfirmed rate was 52% (117/226, Table 1). 35% (33/94) in fresh-frozen sample and 64% (84/132) in FFPE sample were disconfirmed. This indicates a high false-positive rate of WES in detecting low-level variants.

**Table 1:** Summary of WES-detected variant confirmation results by blocker displacement amplification (BDA).

	N	Variant confirmed	Variant absent	Variant misidentified	Disconfirmed rate (%)
FF	94	61	33	0	35
FFPE	132	48	79	5	64
Total tested	226	109	112	5	52
FF (cancer-related)	6	1	5	0	83
FFPE (cancer-related)	60	11	47	2	82
Total tested (cancer-related)	66	12	52	2	82

In the 66 cancer-related variants, the disconfirmed rate was as high as 82% (54/66, Table 1). The confirmed cancer-related variants by this study are listed in Table 2 (Sanger traces in Supplementary Fig. S1-S2). The 12 confirmed variants include 10 substitutions, 1 small deletion (2-bp deletion in dinucleotide repeats) and 1 large deletion (62-bp deletion). Confirmatory results for all 226 variants are listed in Supplementary Table S1.

**Table 2:** WES-detected low-level variants in cancer-related genes confirmed by blocker displacement amplification (BDA).

Sample	DNA variant (GRCh38)	Gene	Protein variant	Category	WES (variant/total reads)
FF	chr12:g.18381739_18381740del	PIK3C2G	–	Intron	3.9% (15/382)
FFPE	chr5:g.1293375G > A	TERT	p.Ser504Leu	Nonsynonymous	1.7% (5/289)
FFPE	chr7:g.2915318G > A	CARD11	p.Arg920Cys	Nonsynonymous	1.4% (4/296)
FFPE	chr8:g.127740689G > A	MYC	p.Glu366Lys	Nonsynonymous	2.1% (4/187)
FFPE	chr9:g.130885328G > A	ABL1	p.Arg1032Gln	Nonsynonymous	3.7% (10/270)
FFPE	chr10:g.8058457G > A	GATA3	p.Val132Ile	Nonsynonymous	1.7% (14/828)
FFPE	chr13:g.109783068C > T	IRS2	p.Gly996Ser	Nonsynonymous	1.4% (5/361)
FFPE	chr19:g.11010434G > A	SMARCA4	p.Arg726His	Nonsynonymous	3.3% (7/209)
FFPE	chrX:g.53194684G > A	KDM5C	p.Arg1165Cys	Nonsynonymous	4.5% (9/198)
FFPE	chr1:g.36466451_36466512del	CSF3R	p.Ser813ProfsTer48	Frameshift	3.5% (3/86)
FFPE	chr15:g.90085380G > A	IDH2	p.Gly325 =	Synonymous	3.1% (7/224)
FFPE	chr19:g.42273661G > A	CIC	p.Ser626 =	Synonymous	2.2% (15/676)

In fresh-frozen sample, variants > 3% VAF show a better confirmed rate (87%), compared to that of variants ≤ 3% VAF (50%). While in FFPE sample, all VAF brackets have a low confirmed rate (overall 36%) (Fig. 3b). This is likely due to the sequencing depth differences. Among the 155 somatic low-level variants from fresh-frozen sample (mean depth 824 ×), we unbiasedly selected 94 variants for BDA analysis. The mean depth for selected variants was 774 × , with minimal depth 95 × . All variants had at least 4 variant reads in WES. Among the 2852 somatic low-level variants from FFPE sample (mean depth 243 ×), we unbiasedly selected 132 variants for BDA analysis. The mean depth for selected variants was 200 × , with minimal depth 43 × . 131/132 variants had at least 3 variant reads in WES. In FFPE sample, the relatively low sequencing depth in the called variants hinders accurate variant calling, thus the reliability of FFPE-called variants is less than in fresh-frozen.

The disconfirmed rates across different variant functional categories were similar (Fig. 3c). For fresh-frozen sample, the disconfirmed rate of nonsynonymous, frameshift, synonymous, intron, and regulatory variants ranges from 32% (nonsynonymous) to 43% (frameshift). For FFPE sample, the disconfirmed rate across different categories ranges from 59% (nonsynonymous) to 77% (regulatory).

The disconfirmed rates by different alteration types varied widely. The A > C/T > G variant had the highest disconfirmed rate in both the fresh-frozen and FFPE samples (Fig. 3d). In the FFPE sample, the C > T/G > A variant accounted for 60% (79/132) of WES-detected variants and 92% (44/48) of the confirmed variants; the disconfirmed rate was significantly lower than other alteration types. The results agree with the fact that the FFPE samples represent cytosine deamination events which cause increased C > T/G > A variants^26–28^.

### BDA qPCR/Sanger uncovering of WES false-negative variants

**Figure 4:**
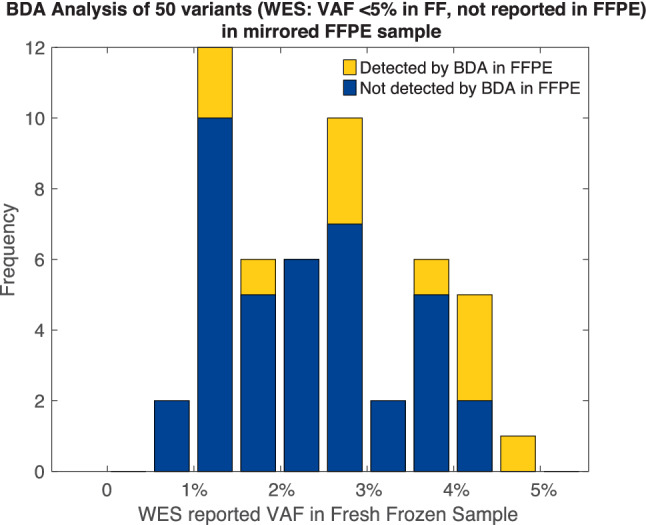
WES false-negative analysis. BDA qPCR/Sanger analysis was performed for 50 variants on the FFPE sample. By WES, those variants were detected with < 5% VAF in the fresh-frozen sample but not detected in the FFPE sample.

Another issue that BDA qPCR/Sanger analysis can address is false-negative variants by WES. Among the 155 low-level (VAF < 5%) variants in the fresh-frozen sample, only 25 were also present in the 8201 FFPE variants. We randomly selected a subset of 50 low-level variants detected by WES in the fresh-frozen sample but not in the mirrored FFPE sample for BDA analysis (Fig. 4). Of these, 39/50 tested negative by BDA and thus were WES true negatives; 11/50 tested positive by BDA in the FFPE sample and thus were WES false negatives.

## Discussion

In this article we describe a study with 226 WES detected variants of VAF < 5% analyzed by BDA qPCR/Sanger method. 52% (117/226) of the variants were disconfirmed. The WES sequencing depth in this article was ~ 1000 × , which was significantly beyond standard practice of 100 × , thus we expect to have higher confirm rate than standard practice. This infers that in standard practice, a higher fraction of low VAF variants needs confirmation. For clinical testing, high sensitivity and specificity of NGS results are essential as clinicians rely on this data to make surveillance and treatment selection. We recommend orthogonal confirmation for low level variants (VAF < 5%) in clinical sample testing. For low quality DNA samples like the FFPE sample used in this study, we recommend confirmation for variants with higher threshold (e.g. VAF < 20%). The fresh-frozen and FFPE samples tested were a mirrored pair from the same NSCLC tumor. In WES identified 8201 somatic variants from the FFPE sample, 2176 variants had VAF > 20% and 6025 variants had VAF < 20%. Among the VAF > 20% variants, 49% (1059/2176) were concordant with the mirrored fresh-frozen sample; while for VAF < 20%, only 0.9% (52/6025) were concordant. This observation again challenges the reliability of variants with WES VAF < 20% in FFPE sample and explains the high disconfirm rate in FFPE sample. Previous NGS confirmation study by Mu et al. also reported that most variants with VAF < 20% were disconfirmed by Sanger^15^.

The BDA allele enrichment + Sanger sequencing method is compatible with any WES hybrid capture panel and bioinformatics pipeline. As a demonstration we chose Agilent hybrid capture panel + Illumina DRAGEN somatic small variant caller. For our ultra-large dataset (> 1000 × WES) without matched normal samples, Illumina DRAGEN bioinformatics pipeline was chosen based on high accuracy, high efficiency, and for not needing matched normal samples^29,30^. A recent benchmark study showed that across 5 datasets, DRAGEN produces 14–67% and 22–91% fewer false SNV calls, 35–86% and 48–89% fewer false indel calls, at 75% and 830% faster speed than Strelka2 and Mutect2 respectively^24^. Calling variants for WES at < 5% VAF is extremely challenging and the sensitivities of bioinformatics pipelines vary based on depth and tumor purity^31^. To limit BDA + Sanger confirmation of WES variants and save time and resources, researchers could potentially analyze the sequencing data using multiple aligners and callers to rule out obvious false positives, and only select a suspicious subset for downstream orthogonal confirmation.

By adopting BDA technology, we can reliably report mutations with VAF as low as 0.1%, significantly improving clinical sensitivity without sacrificing clinical specificity in tumor samples or blood plasma cfDNA. Coupled with BDA technology, the heightened sensitivity in Sanger sequencing allows for confirmation of low-level variant identity without additional steps. In cases where VAF is not needed, BDA PCR can be implemented on standard thermocyclers. Digital PCR also strives to capture low-level variants^32,33^; however, if the actual mutation is neither wildtype nor a putative variant (e.g., WES-misidentified cases in this manuscript), digital PCR would fail to detect the mutation because the probes target the putative variant. In contrast, BDA enriches any alleles different from the wildtype and is not narrowly variant specific. Additionally, digital PCR requires specialized equipment and synthesis of multiple dual-labeled TaqMan probes. BDA technology only uses non-modified, non-HPLC-, non-PAGE-purified DNA oligos and does not need chemically functionalized DNA probes for detection, therefore reduces the turnaround time for oligo synthesis and customized variant confirmation.

BDA allele enrichment + Sanger sequencing provides a time-efficient and cost-effective option for determining low-frequency gene mutations. It only takes 2–3 days from BDA design to results. All oligos are ordered as non-modified, non-purified standard oligos and delivered the next day. After 1.5 h of BDA qPCR analysis, the amplicons are sequenced either by the lab itself (to get the results the same day as qPCR) or sent to a service provider or an institutional core facility (to get the results the next day).

In addition to oncology applications, BDA high sensitivity detection can be utilized in any areas where low VAF DNA variant analysis is needed. One existing application is genetic mosaicism detection^18,20,34,35^. Other potential applications include monitoring of chimerism in patients after stem cell transplantation^36^, analysis of mitochondrial heteroplasmy in mitochondrial diseases^37,38^, rare microbe detection for microbiome profiling^39^ and confirmation of metagenomic sequencing results^40^.

## Supplementary Information

Supplementary Figures

Supplementary Table.

Supplementary Table Legend.

## Data Availability

The datasets generated and/or analyzed during the current study are available from the corresponding author on reasonable request.
