# Supplementary material for: Confirming putative variants at ≤ 5% allele frequency using allele enrichment and Sanger sequencing
Source: Sci Rep. 2021 Jun 2;11:11640. doi: 10.1038/s41598-021-91142-1 (PMC8172533; doi:10.1038/s41598-021-91142-1)

Confirming putative variants at ≤5% allele frequency using allele enrichment and Sanger sequencing

Yan Helen Yan[1], Sherry X. Chen[2], Lauren Y. Cheng[2], Alyssa Y. Rodriguez[1], Rui Tang[1], Karina Cabrera[1] and *David Yu Zhang[2,3]

[1]NuProbe USA, Inc., Houston, TX

[2]Department of Bioengineering, Rice University, Houston, TX

[3]Systems, Synthetic, and Physical Biology, Rice University, Houston, TX

Corresponding author: David Yu Zhang**;** 6500 Main St, Houston, TX, 77030**;** (713) 348-2832**;** dyz1@rice.edu

Supplemental Figure 1.

Sanger traces for WES-detected low-level variants in cancer-related genes confirmed by blocker displacement amplification (BDA)

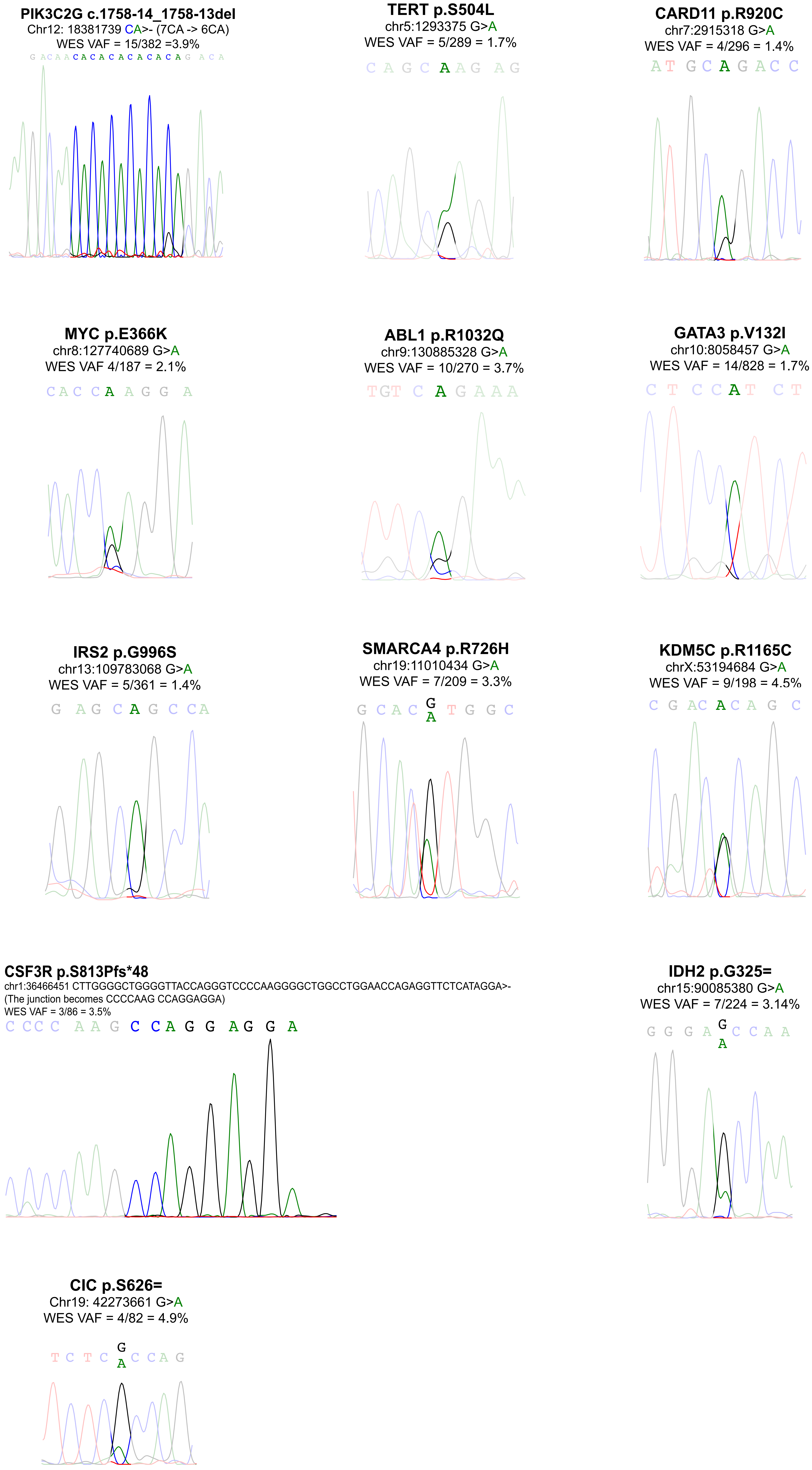

Supplemental Figure 2.

Sanger traces for selected WES-detected low-level variants in cancer-related genes disconfirmed by blocker displacement amplification (BDA) (Sanger traces show wildtypes rather than WES called variants)

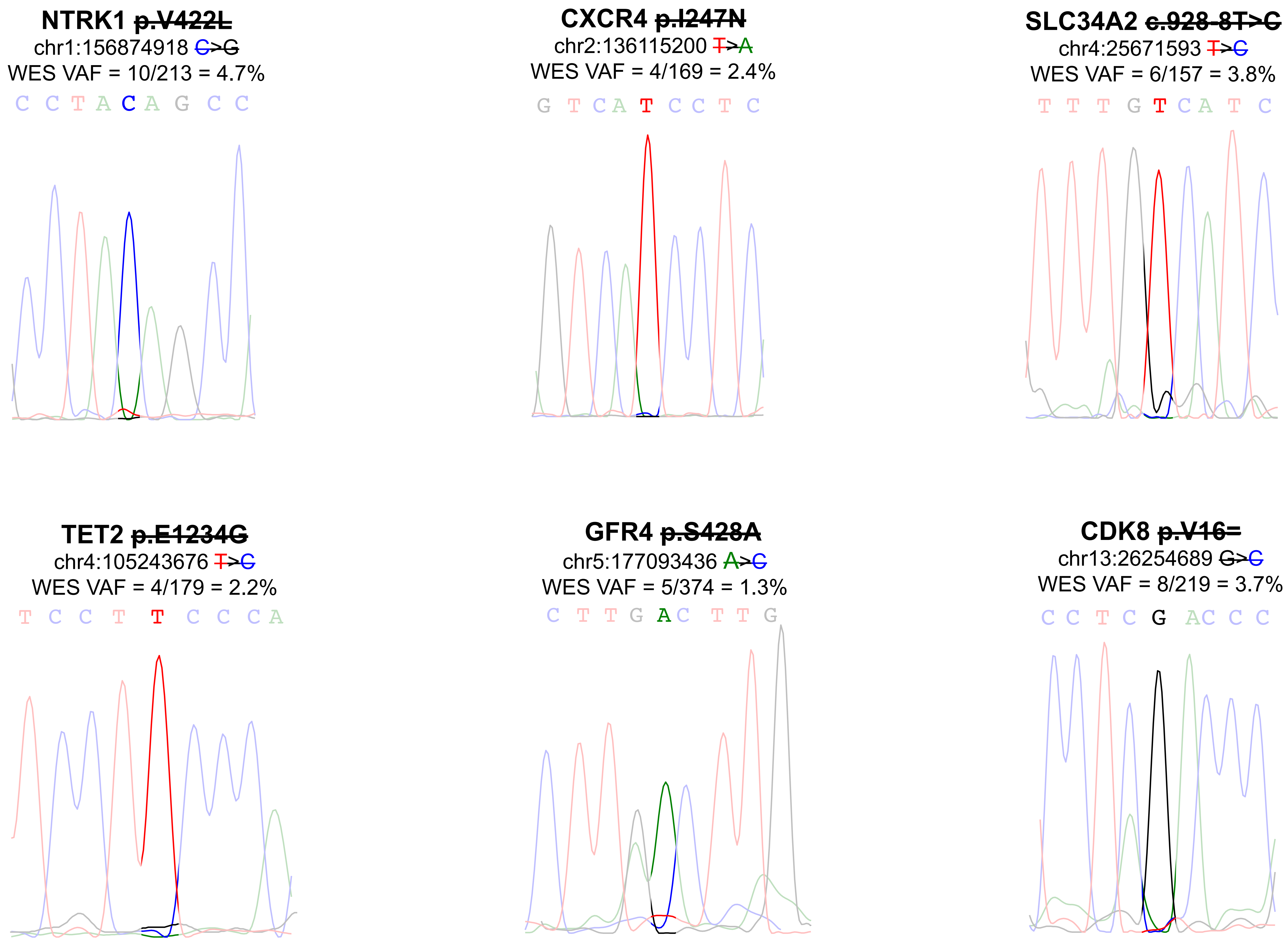

Supplemental Figure 3.

5 WES-misidentified low-level variants

(Sanger traces after BDA show neither wildtypes nor putative variants; Amplicon-based NGS confirmed BDA results)

Whole Exome Sequencing Results	BDA Results	Amplicon-based NGS Results
Chr9:33922699 G>A	Chr9:33922699_33922700insA	Chr9:33922699_33922700insA
VAF = 4/238 = 1.7%	VAF = 0.87%	VAF = 107/9134 = 1.2%

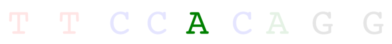

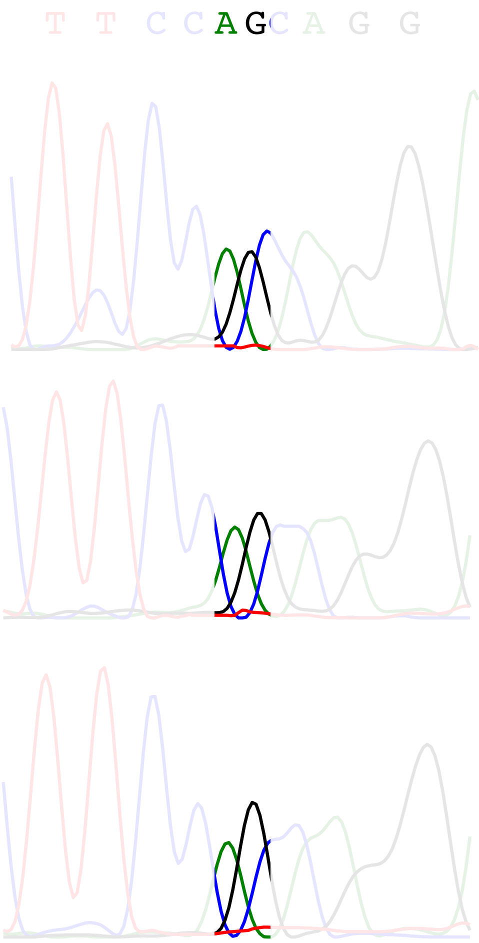

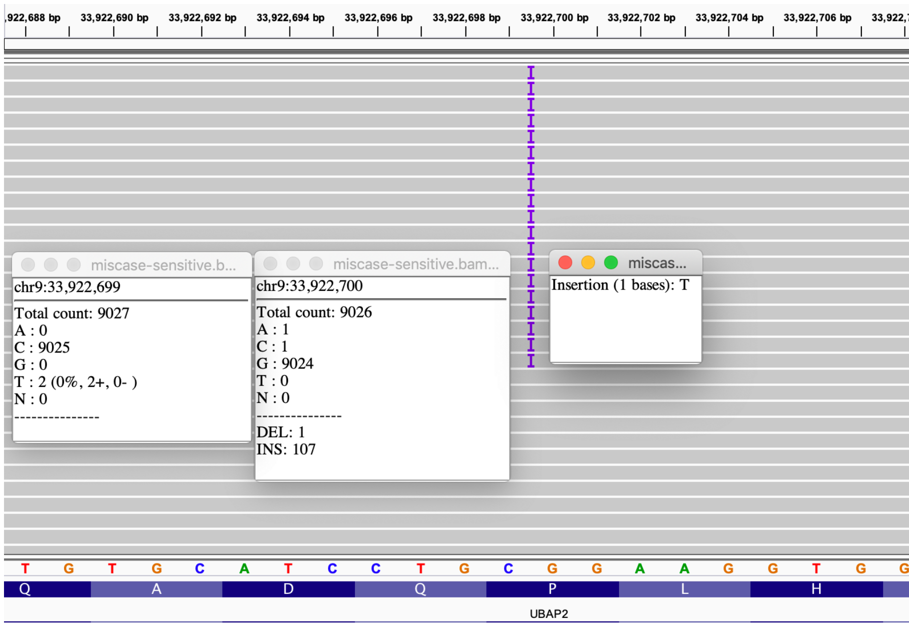

Whole Exome Sequencing Results

chr11:1952119 C>A

VAF = 1/43 = 2.3%

G A G G A G A A G

BDA Results

chr11:1952119 C>T

VAF = 0.32%

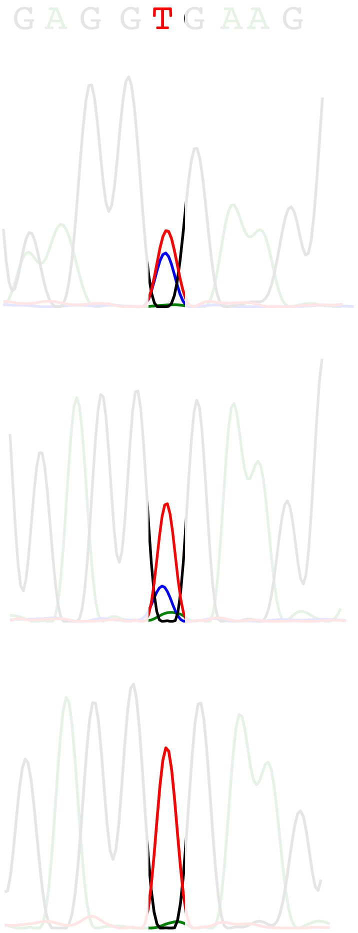

Amplicon-based NGS Results

chr11:1952119 C>T

VAF = 10/1892 = 0.5%

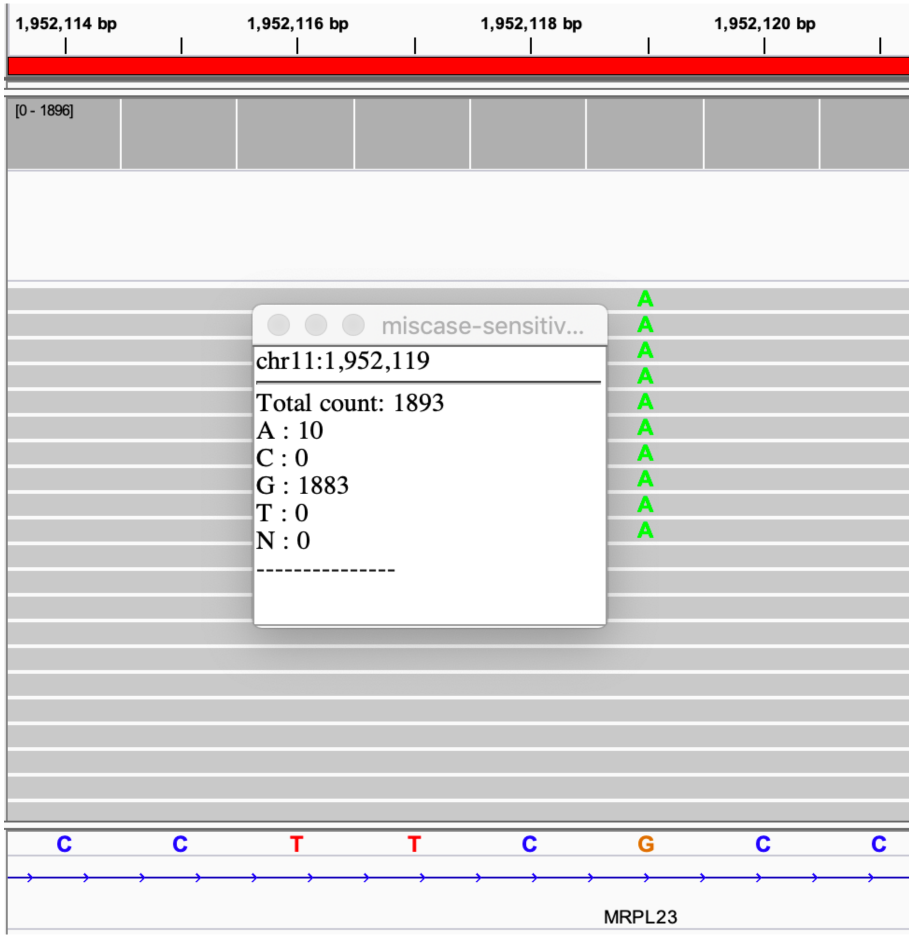

Whole Exome Sequencing Results

chr18:76988457 G>A
VAF = 8/173 = 4.6%

T T C C A T C C C

BDA Results

chr18:76988457_ 76988458insA
VAF = 0.61%

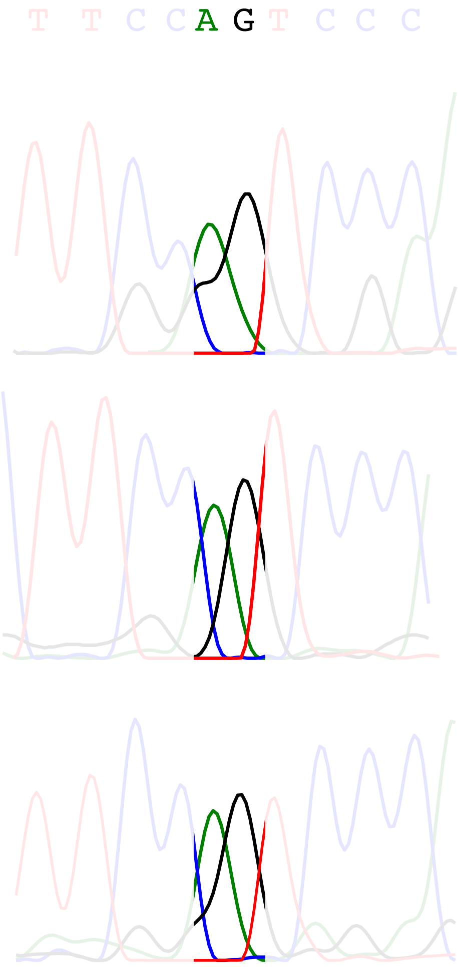

Amplicon-based NGS Results

chr18:76988457_ 76988458insA
VAF = 47/2518 = 1.9%

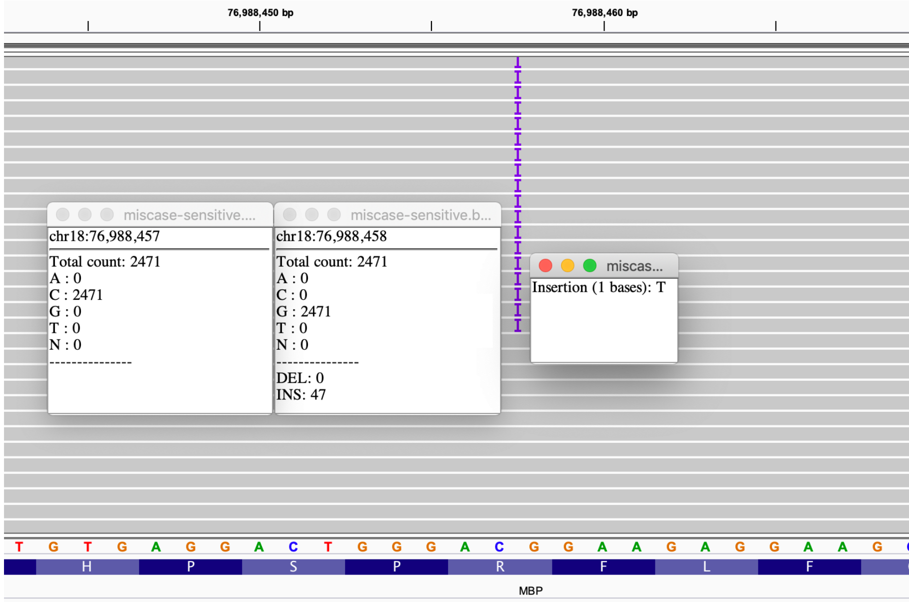

Whole Exome Sequencing Results

chr7:2944337 G>A

VAF = 4/332 = 1.2%

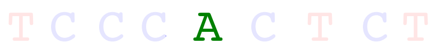

BDA Results

chr7:2944336_2944337insA

VAF = 0.27%

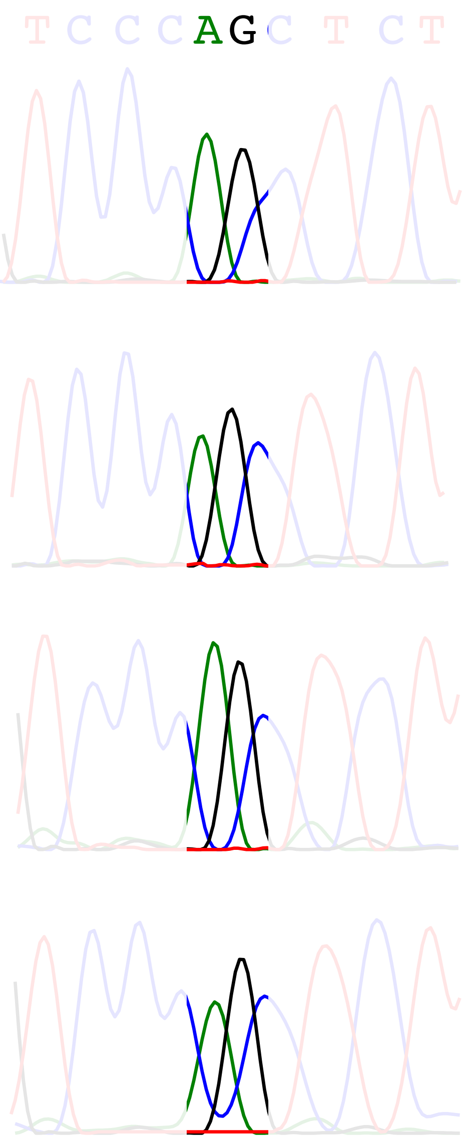

Amplicon-based NGS Results

chr7:2944336_2944337insA

VAF = 129/8640 = 1.5%

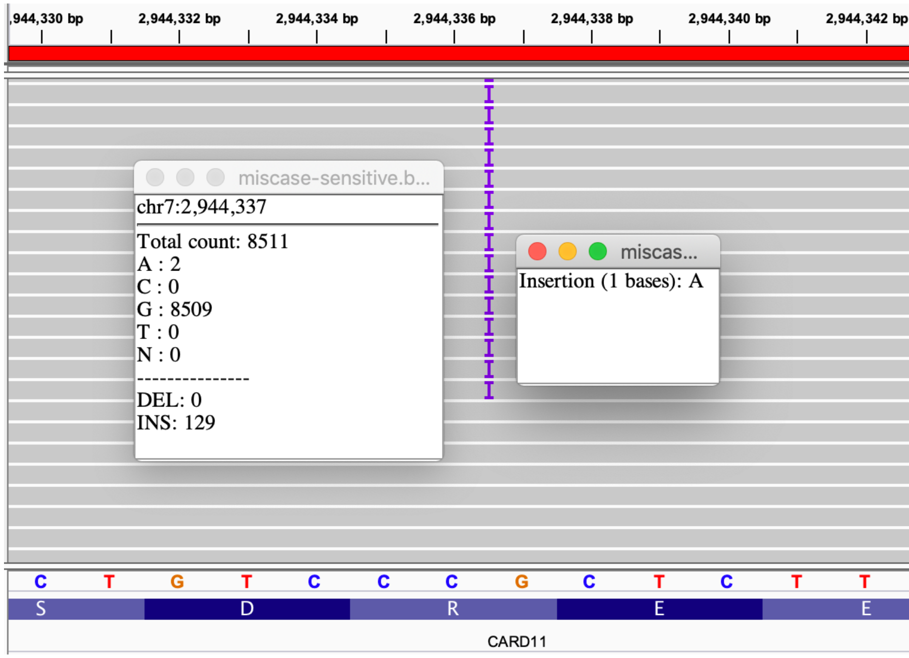

Whole Exome Sequencing Results

chr16:2084656 G>C
WES VAF = 6/150 = 4.0%

AG GCCTC CC

BDA Results

chr16:2084656 G>A
VAF = 0.23%

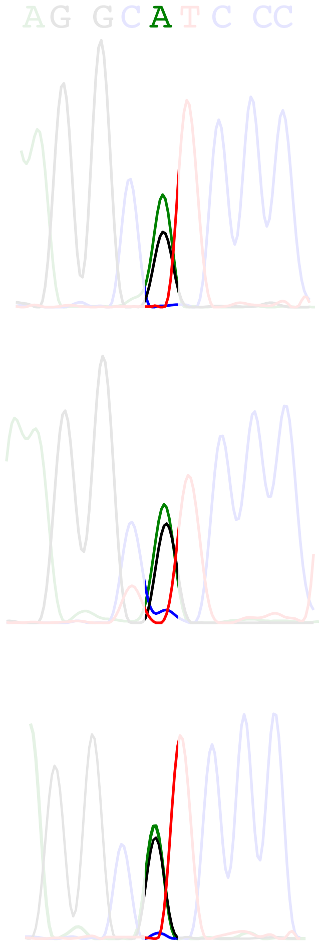

Amplicon-based NGS Results

chr16:2084656 G>A
VAF = 17/4227 = 0.4%

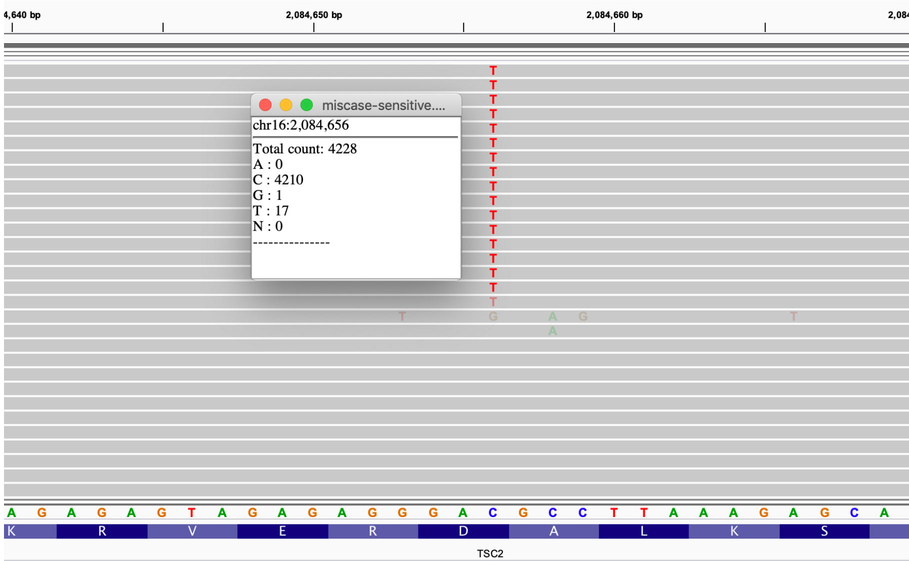

Supplement: Supplementary file 1 — Supplementary Figures [file 41598_2021_91142_MOESM1_ESM.pdf]
