# Supplementary material for: Confirming putative variants at ≤ 5% allele frequency using allele enrichment and Sanger sequencing
Source: Sci Rep. 2021 Jun 2;11:11640. doi: 10.1038/s41598-021-91142-1 (PMC8172533; doi:10.1038/s41598-021-91142-1)
Supplement: Supplementary file 3 — Supplementary Table Legend. [file 41598_2021_91142_MOESM3_ESM.docx]

**Supplementary Table S1.** Confirmatory results for all 226 variants. GRCh38 based genomic coordinate, protein and CDS (coding sequence) variant, WES reads and BDA measured VAF are included for each variant.
